# Biochar-cadmium retention and its effects after aging with Hydrogen Peroxide (H_2_O_2_)

**DOI:** 10.1016/j.heliyon.2021.e08476

**Published:** 2021-11-26

**Authors:** Bárbara Samartini Queiroz Alves, Luiz Arnaldo Fernandes, Randal J. Southard

**Affiliations:** a Department of Land, Air and Water Resources, University of California-Davis, Davis, CA 95616, USA; b Institute of Agrarian Sciences, Federal University of Minas Gerais, Montes Claros, MG 39404547, Brazil

**Keywords:** Biochar, Adsorption, Aging with H_2_O_2_, Cd remediation, Soil chemistry, Waste management

## Abstract

Cadmium (Cd) is a highly toxic heavy metal that can become available to the environment from a variety of sources. The thermal transformation of organic residues into biochar can be a sustainable way to reduce cadmium environmental availability and, at the same time, a waste management solution. We studied sixteen biochars in two versions: unaged and aged with hydrogen peroxide (H_2_O_2_), regarding their Cd retention capacity. Feedstocks used included softwood biochar (SWB), almond shell (ASB), walnut shell (WSB), sewage sludge (SSB), and coconut shell (CSB); production temperatures varied from 450 to 900 °C. The objectives of this research were to understand the role of biochar properties on Cd adsorption rates and to evaluate how properties and adsorption rates vary as a function of H_2_O_2_ aging. Feedstock played a more important role than production temperature in determining biochar properties. Cd-adsorption capacity ranged from 0.67 to 415.67 mg/g, and the biochars that adsorbed the most Cd were SSB 700, SWB 800 – i, CSB 600 – m2, ASB 500–1, CSB 600 – m3, WSB 900, and CSB 600. The properties that best explained this variation in Cd retention were ash, sulfur, nitrogen and carbon content. Variation in oxygen content, cation exchange capacity and surface area had less impact of Cd adsorption. The H_2_O_2_ aging caused oxygen content to increase in all biochars, but the increase in Cd retention was not significant for the majority of the biochars and aging even reduced the Cd retention in some. Our results may help design biochars with maximized sites for Cd adsorption.

## Highlights

• Biochars-Cd adsorption capacity varied from 0.67 to 415.67 mg/g.
• Feedstock seems to play a more important role than temperature when predicting Cd retention.
• Ash, sulfur, nitrogen and carbon content were the most important properties for predicting Cd retention.

## 1 Introduction

Cadmium (Cd) is a highly toxic heavy metal that can be released to the environment from natural sources such as volcanic activity, weathering of Cd-containing rocks, and sea spray or from anthropogenic activities including manure and phosphorus fertilizer application, mining, smelting, refining and electroplating activities (Roberts 2014). When present in water and soil, plants can accumulate Cd in their tissues (Kim et al., 2019) and when consumed by humans it can cause health issues such as renal dysfunction, osteoporosis and cancer (Bashir et al., 2014). Therefore, it is imperative to find ways to reduce the availability of Cd in soils, preventing its uptake by plants and their subsequent consumption by humans and animals.

Cadmium behavior in the soil solution depends on the degree of acid “hardness” or “softness”, as defined by Pearson (1963). This concept can be utilized to distinguish Cd from nutritive cations that would otherwise compete for surface functional groups. Explicitly, Cd is a soft acid, i.e., an electron acceptor atom of low positive charge and large size and has several easily excited outer electrons. A soft acid is polarizable and covalently binds to soft bases. Soft sites are N- and S-containing sites (amine, amide and thione), whereas hard base sites are attributed to oxygenated functional groups (Sparks 2003). This behavior can be explained by π-bonding theory and electron correlation theory. Hard acids have tightly held outer electrons, but there are also empty orbitals at low available energy levels. Atoms such as O and F could form π-bonds by donating electrons from the ligand to the empty orbitals of the metal. In contrast, Cd exhibits a repulsive interaction between the two sets of filled orbitals on the metal and O and F ligands (Pearson 1963). Many methods have been used to remove Cd from the aqueous phase, such as treatment of potable water, including chemical precipitation, coagulation-flocculation, flotation, ion exchange, membrane separation (Kurniawan et al., 2006) and adsorption (Liu et al., 2019). Among these methods, adsorption using low-cost materials has received much attention recently due to their cost effectiveness, ability to reuse materials that might otherwise be discarded, and the potential for these materials to sorb a wide range of pollutants (Park et al., 2017). Examples of low-cost sorbents include clay minerals (Wang et al., 2020a), biomass, agricultural wastes, activated carbon, and biochar (Ulmanu et al., 2003). The use of biochar has been gaining interest because it can be produced using clean technologies and can serve as a waste management solution (Jeffery et al., 2015; Tan et al., 2015).

Biochar is a carbon (C) rich porous material produced by thermochemical break down of organic materials in a low oxygen or anoxic environment, thus preventing combustion (Lehmann and Joseph 2009). Common production methods are gasification, pyrolysis, hydrothermal (Kwapinski 2019) and thermal carbonization (López et al., 2020). All methods require heating of the biomass feedstock. The difference between methods is the quantity of oxygen allowed in the system, temperature ranges, water content, and pressure (Pecchi and Baratieri 2019). Depending on the production conditions and feedstock, the physical and chemical characteristics of biochars can vary widely, resulting in biochars with different types and quantities of surface functional groups, surface area, pH, and chemical composition (Harsono et al., 2013; Laird et al., 2009). Due to their surface characteristics, including a porous structure, high surface area, and carbonized and non-carbonized domains, biochar is capable of adsorbing heavy metals (Inyang et al., 2016; Mohan et al., 2014) and a variety of organic pollutants in the aqueous phase (Dai et al., 2019; Huang et al., 2019).

The vast literature on biochar-metal retention has confirmed four mechanisms for Cd sorption on biochars: (i) cation exchange (e.g., Ca^2+^, Mg^2+^, K^+^, Na^+^) with Cd^2+^; (ii) surface precipitation with anions (e.g., PO_4_^3-^, CO_3_^2-^ and SiO_3_^2-^); (iii) surface complexation between Cd^2+^ and polar functional groups (e.g., –OH, C=O, C–O) and (iv) physical sorption by Cd^+2^ coordination with π electrons on the biochar graphene-like structure (e.g. C=C, C≡C) (Li et al., 2017a, Li et al., 2017b; Tan et al., 2015). Understanding the role of each of these mechanisms is important to predict biochar's potential for Cd removal.

Additionally, even though biochar is known for being highly recalcitrant (Ahmad et al., 2014), as an organic material, once placed in the soil it will be susceptible to natural aging (Wang et al., 2016), which can affect its ability to immobilize Cd in soil (Nagodavithane et al., 2014; Xu et al., 2018). Common changes are: 1) oxidation due to microbial attack of the labile domain, plant acidic exudates, and chemical weathering; and 2) physical breakdown due to wetting-drying and freezing-thawing cycles that it may undergo (Mia et al., 2017a; Xu et al., 2018). The reported results show that when biochar ages it usually becomes more acidic (Cheng and Lehmann 2009; Zhao et al., 2015), increasing the CEC and number of oxygenated surface functional groups (e.g. carboxylic acids) (Yao et al., 2010), while lowering surface area and aliphatic C (Ghaffar et al., 2015; Wang et al., 2017).

Research methods for studying aging effects on biochars include: composting (Wiedner et al., 2015), incubations (Mukome et al., 2014; Singh et al., 2016), field (Khorram et al., 2017; Mukherjee et al., 2014; Ren et al., 2018), application of dry-wet and freeze-thaw cycles (Xu et al., 2018), application of hot air (Wang et al., 2017), acid leaching with HNO_3_/H_2_SO_4_ (Hadjittofi et al., 2014; Lu et al., 2015; Qian et al., 2015), and alkaline leaching with KOH (Goswami et al., 2016; Jin et al., 2014; Petrović et al., 2016), NaOH (Pietrzak et al., 2014) or H_2_O_2_ (Huff and Lee 2016; Lawrinenko et al., 2016; Mia et al., 2017b; Xue et al., 2012; Zuo et al., 2016). We only found one research paper with the specific focus on evaluating how Cd adsorption changes after aging with H_2_O_2_. Frišták et al. (2015) aged biochars made from garden green waste residues with H_2_O_2_ and noticed an increased Cd retention; however, it is not yet fully understood how biochar-Cd retention would change after oxidation. We believe this research also innovates with utilization of H_2_O_2_ as an aging proxy for assessing how Cd retention would change over time.

The objectives of this research were to: (i) understand the role of biochar surface functional groups, surface area, ash, and CEC on Cd adsorption rates by elucidating specific retention mechanisms, and (ii) evaluate how biochar adsorption rates of Cd change after aging biochar with H_2_O_2_. We hypothesize: (i) biochars with greater soft bases (S + N) functional group content will bind Cd more effectively than biochars that have higher O-containing functional groups; (ii) for biochars poor in S and N functional, carbon content will play a bigger role in Cd retention than surface area and CEC; (iii) aging biochars will increase their Cd retention. Information from this study may help to predict Cd retention and help design biochars with increased sorption capacity.

## 2 Materials and methods

### 2.1 Biochar acquisition

Fourteen commercial biochars were donated by six different companies and two biochars were made from sewage sludge at the Biomass Laboratory at UC Davis (Table 1). Companies were identified by letters to prevent their exposition.

**Table 1:** Biochars experimental matrix and description of production methods.

#	Sample ID	Feedstock	Temp. (^o^C)	Production method	Production Company	Residence time	Post production modification
1	SWB 800 - i	Softwood	800	Gasification	A	2 min	Yes, inoculation. Information on Margenot et al. (2018)
2	SWB 650	Softwood	650	Slow pyrolysis	B	3 Days	None
3	SWB 650 – m1	Softwood	650	Slow pyrolysis	B	3 Days	Yes, proprietary information
4	SWB 500	Softwood	500	Fractional Hydro Pyrolysis	C	20 min	None
5	ASB 500 - 1	Almond Shell	500	Fast pyrolysis	D	45min	None
6	ASB 500 - 2	Almond Shell	500	Fractional Hydro Pyrolysis	C	20 min	None
7	WSB 500	Walnut Shell	500	Fast Pyrolysis	D	45 min	None
8	WSB 900	Walnut Shell	900	Gasification	E	2 min	None
9	WSB 700 - 60	Walnut Shell	700	Thermal carbonization under 60 psi of pressure	F	50 min	None
10	WSB 700 - 90	Walnut Shell	700	Thermal carbonization under 90 psi of pressure	F	50 min	None
11	SSB 450	Sewage Sludge	450	Slow pyrolysis	UC Davis Lab	5 h	None
12	SSB 700	Sewage Sludge	700	Slow pyrolysis	UC Davis Lab	5 h	None
13	CSB 600	Coconut Shell	600	Slow pyrolysis	B	3 Days	None
14	CSB 600 - m1	Coconut Shell	600	Slow pyrolysis	B	3 Days	Yes, proprietary information
15	CSB 600 - m2	Coconut Shell	600	Slow pyrolysis	B	3 Days	Yes, proprietary information
16	CSB 600 - m3	Coconut Shell	600	Slow pyrolysis	B	3 Days	Yes, proprietary information

Each biochar sample was mixed, air dried to constant mass, manually ground with mortar and pestle, and passed through a 2 mm sieve for subsequent analysis.

### 2.2 Biochar characterization

Electric conductivity and pH were determined by a dilution of 1:20 biochar with reverse osmosis (RO) H_2_O (w:v) and equilibration for 90 min on a vertical shaker, according to Rajkovich et al. (2012), and following IBI recommendation (IBI 2015). Total C, N, O, H and S analysis were conducted with a dry combustion-elemental analyzer Costech ECS 4010 instrument. Moisture, volatile matter (VM), and ash were determined following ASTM D1762-84 Standard Test Method for Chemical Analysis of Wood Charcoal (ASTM 2013). Dissolved organic carbon (DOC) was determined after column RO water extractions and measured with a Shimadzu Total Organic Carbon Analyzer (TOC-V CSH). Cation exchange capacity (CEC) was determined with a method adapted from Mukome et al. (2013) using a first extraction with 1 mol L^−1^ sodium acetate (pH 8.2), following by 2-propanol washes and 1 mol L^−1^ ammonium acetate (pH 7) extractions. The sodium (Na) content in the leachate was measured with Atomic Absorption Spectrophotometer PerkinElmer 4100ZL. Total pore area and volume were determined with CO_2_ gas adsorption with a degasification step under 120 °C, following recommendations of Sigmund et al. (2017), calculated by Density Functional Theory (DFT) pore size theory, and provided by Micromeritics® Analytical Laboratory following ISO (2010). For particle size distribution samples were measured by a laser diffraction method according to Eshel et al. (2004) using a Coulter LS-230 Particle Size Analyzer. Samples were milled and oven dried for the determination of functional groups using a Thermo Scientific Nicolet 6700 spectrometer to obtain diffuse reflectance infrared spectra (DRIFTS).

### 2.3 Biochar aging (oxidation)

To investigate aging effects on biochar capacity for retaining Cd, 30 g of biochars were placed in a 1L plastic bottle with 600mL of H_2_O_2_ (30%). The bottles were left open overnight, then they were closed and placed on an orbital shaker for 2 h at room temperature (120 rpm). After that, biochar solutions were separated by vacuum filtration, washed with 600 mL of RO water and air dried at room temperature. For practical reasons, this experiment was conducted only with the seven biochars that retained the most Cd.

### 2.4 Biochar-Cd adsorption experiments

Batch adsorption experiments were carried out by adding 0.48g ± 0.005g (air-dry weight basis) of biochar to 15 mL plastic centrifuge tubes with 12 mL of solution (1:25 solid/solution ratio) with six concentrations of CdCl_2_ (0, 12, 25, 50, 100, 200 mg L^−1^) and 5 mmol L^−1^ NaCl, following the methods of Bair et al. (2016). Prior to running the adsorption Cd experiments, two sets of samples (no replicates for the concentrations) were used to determine the necessary adjustment to pH 7, using 0.1 or 1 mol L^−1^ NaOH, or 0.1 or 1 mol L^−1^ HCl depending on the biochar-solution pH measured after a 24 h reaction time using an end-over-end shaker at 8 rpm. The first set was used to make a gross adjustment of the pH, and the second set was used to make a fine adjustment. The final volume of acid or base added was recorded and used to titrate the third and real batch prior to shaking.

The adsorption batch experiments were conducted in three replicates for each concentration of Cd, after shaking for 24 h at room temperature on an end-over shaker at 8 rpm. The tubes were then centrifuged for 10 min at 8,000 × g, and the supernatant filtered with a Fisher Scientific nylon syringe filter with 0.45 μm pore size. The pH of the extracted solution was measured immediately to verify the target pH, and then the solution was acidified (pH < 2) for preservation using a 10 mol L^−1^ HCl solution. Cd concentrations were quantified using atomic absorbance spectrometry (AAS) using a graphite furnace (PerkinElmer Analyst 800) with a lamp at 228.8 nm wavelength for Cd. Calibration curves were made by serial dilution of 1000 mg L^−1^ atomic absorption reference standard solutions (Fisher Scientific) with an identical background solution of 5 mmol L^−1^ NaCl used in experimental samples.

The biochar-Cd removal percentage (R) and adsorption capacity at equilibrium (q_e_) were calculated using the following equations:

(1) $$\text{R}=\frac{{\text{C}}_{0}\;–{\text{ ​C}}_{\text{e}}}{{\text{C}}_{0}}\times 100\%$$

(2) $${\text{q}}_{\text{e}}=\frac{\left({\text{C}}_{0}–{\text{ ​C}}_{\text{e}}\right)\text{ ​V}}{\text{W}}$$

where *C*_*0*_ and *C*_*e*_ were the initial and the equilibrium concentrations of Cd in mmol L^−1^, *V* was the solution final volume, and *W* was the air-dried mass of biochar in kg.

### 2.5 Adsorption models

To investigate the biochar-Cd adsorption mechanism two equilibrium isotherm mathematical models were employed: Langmuir and Freundlich. The Langmuir model, originally derived to describe gas adsorption, assumes that adsorption occurs at specific and identical sites on a homogeneous surface. The adsorbate forms a monolayer until an adsorption maximum is achieved when the surface runs out of sites. The Freundlich model is not theoretically derived and is usually utilized to describe a complex system; it is an empirical equation that accounts for the amount of adsorbate at equilibrium (*q*_*e*_) in relation to its concentration in solution (*C*_*e*_) (Essington 2004). The Freundlich model predicts that the adsorbent does not run out of sites, allowing for infinite surface coverage and no adsorption maximum (Reed and Matsumoto 1993). They can be represented as:

(3) $$q_{e}=\;K_{F}C_{e}^{\frac{1}{n}}\;\text{Freundlich}$$

(4) $$q_{e}=\;\frac{q_{max}\;k_{L\;}C_{e}}{1+\;k_{L}\;C_{e}}\;\text{Langmuir}$$

Where *q*_*e*_ is the amount of Cd adsorbed at equilibrium (mmol kg^−1^) or adsorption capacity, *C*_*e*_ is the equilibrium concentration of Cd found in the solution (mmol/L) and for the Langmuir model *q*_*max*_ is the maximum adsorption capacity (mmol L^−1^), and *k*_*L*_ (L mol^−1^) is the adsorption equilibrium constant that measures the intensity of the adsorption. The same parameters are used for the Freundlich model (*C*_*e*_ and *q*_*e*_) only adding *K*_*F*_ (kg L^−1^) and *1/n* (unitless) that are positive adjustable parameters, not interpreted as having physical meaning, and are related to adsorption capacity and adsorption intensity, respectively. The fitness of each model was verified by comparison of coefficients of determination R^2^.

### 2.6 Statistical analysis

For analyzing the correlation between characterization properties and Cd retention, a factor analysis with dimension reduction (because the sample size is under 30, and thus to obtain more degrees of freedom) by principal components with maximum variance rotation was conducted. After selecting the main components, a linear regression analysis was conducted to correlate variables with Cd retention. Statistical analyses were performed using JMP v.14.0.

## 3 Results and discussion

### 3.1 Biochar characterization

Results from the characterization of biochars are presented in Table 2. The majority of biochars were commercially available and donated from companies, except for sewage sludge biochars that were produced with the intention of maximizing Cd retention. We had special interest in this feedstock because sludge is usually rich in N and S and could be a good alternative for both the managers of the Monterey Wastewater Treatment Plant (MWWTP) where we sampled, and the Salinas Valley, California, USA (a known area with soils enriched in Cd) farmers who would have a local source of biochar.

**Table 2:** Biochar properties.

#	Sample	pH	EC	Moist.	VM	Ash	C	H	O	N	S	DOC	CEC	Pore area	Pore volume
			mS cm^−1^	%								mg L^−1^	cmol_c_ kg^−1^	m^2^ g^−1^)	cm^3^ g^−1^
1	SWB 800 – i	10.57 ± 0.01	2.48 ± 0.04	14.61 ± 0.12	20.50 ± 0.28	16.62 ± 0.19	63.36 ± 0.34	1.07 ± 0.06	14.29 ± 0.01	0.64 ± 0.01	0.91 ± 0.06	187.83 ± 14.51	176.65 ± 2.95	363.62	0.08
2	SWB 650	6.88 ± 0.07	0.20 ± 0.01	4.60 ± 0.10	23.70 ± 0.72	2.80 ± 0.09	82.71 ± 0.37	2.73 ± 0.15	10.17 ± 0.22	0.11 ± 0.00	<0.02%	245.54 ± 23.54	97.65 ± 6.09	281.03	0.06
3	SWB 650 – m1	7.92 ± 0.01	0.14 ± 0.00	21.37 ± 0.56	22.34 ± 0.19	3.09 ± 0.19	65.96 ± 2.59	2.73 ± 0.01	10.18 ± 0.13	0.18 ± 0.04	<0.02%	98.08 ± 9.13	109.13 ± 3.34	305.66	0.07
4	SWB 500	9.54 ± 0.01	1.11 ± 0.02	6.25 ± 0.05	30.56 ± 0.38	2.84 ± 0.09	70.89 ± 0.22	3.55 ± 0.05	17.07 ± 0.48	0.17 ± 0.01	<0.02%	227.70 ± 4.51	16.46 ± 0.32	93.48	0.02
5	ASB 500 - 1	10.52 ± 0.03	3.02 ± 0.04	21.31 ± 0.04	28.21 ± 0.18	10.63 ± 0.04	64.69 ± 0.51	2.42 ± 0.19	16.15 ± 0.15	0.94 ± 0.00	<0.02%	505.24 ± 19.27	115.11 ± 6.45	253.86	0.05
6	ASB 500 - 2	10.17 ± 0.01	3.39 ± 0.03	8.03 ± 0.09	23.25 ± 0.24	13.09 ± 0.72	65.80 ± 0.39	3.06 ± 0.17	17.11 ± 0.61	0.76 ± 0.01	<0.02%	200.43 ± 7.25	24.02 ± 0.47	54.65	0.01
7	WSB 500	10.20 ± 0.04	0.67 ± 0.02	3.97 ± 0.02	24.23 ± 0.17	4.98 ± 0.10	79.88 ± 0.53	2.24 ± 0.28	13.51 ± 1.53	0.72 ± 0.02	<0.02%	142.24 ± 7.70	63.44 ± 2.38	270.85	0.06
8	WSB 900	10.68 ± 0.03	6.02 ± 0.04	2.40 ± 0.12	11.80 ± 0.28	11.60 ± 0.19	78.49 ± 0.84	0.62 ± 0.18	11.88 ± 0.18	0.65 ± 0.01	0.50 ± 0.01	118.79 ± 7.00	203.57 ± 14.95	399.66	0.07
9	WSB 700 - 60	9.16 ± 0.02	1.14 ± 0.04	5.84 ± 0.01	7.83 ± 0.16	3.46 ± 0.16	85.75 ± 0.14	1.68 ± 0.02	6.61 ± 0.14	0.39 ± 0.01	<0.02%	37.74 ± 0.71	164.08 ± 6.82	372.25	0.08
10	WSB 700 - 90	8.62 ± 0.01	0.76 ± 0.01	5.42 ± 0.04	8.97 ± 0.17	3.37 ± 0.10	86.96 ± 0.74	1.89 ± 0.03	6.68 ± 0.36	0.55 ± 0.00	<0.02%	145.11 ± 6.13	145.62 ± 4.52	310.39	0.07
11	SSB 450	6.47 ± 0.03	2.84 ± 0.01	2.90 ± 0.07	25.30 ± 0.90	59.69 ± 0.10	23.31 ± 0.35	1.45 ± 0.04	15.04 ± 0.41	3.25 ± 0.05	3.72 ± 0.06	5.18 ± 0.03	53.92 ± 1.47	70.77	0.01
12	SSB 700	12.53 ± 0.01	6.86 ± 0.19	2.61 ± 0.03	9.74 ± 0.36	74.14 ± 0.58	25.99 ± 0.53	0.69 ± 0.02	11.25 ± 0.05	1.54 ± 0.03	6.45 ± 0.12	10.60 ± 0.11	44.96 ± 1.13	75.07	0.02
13	CSB 600	8.02 ± 0.00	0.51 ± 0.00	8.52 ± 0.09	30.84 ± 1.47	7.0 ± 0.25	70.64 ± 0.25	3.35 ± 0.26	16.20 ± 0.05	0.58 ± 0.01	0.49 ± 0.02	211.12 ± 18.34	84.49 ± 2.55	244.07	0.05
14	CSB 600 - m1	7.71 ± 0.02	0.15 ± 0.01	9.38 ± 0.03	30.57 ± 0.60	3.28 ± 0.06	67.46 ± 0.61	3.15 ± 0.10	18.56 ± 0.45	0.47 ± 0.03	0.25 ± 0.02	86.62 ± 8.26	53.82 ± 1.11	233.65	0.05
15	CSB 600 - m2	7.05 ± 0.01	0.72 ± 0.01	35.58 ± 0.27	35.00 ± 0.44	12.45 ± 0.02	53.96 ± 0.16	3.03 ± 0.20	19.23 ± 0.52	0.67 ± 0.03	0.69 ± 0.01	121.88 ± 7.28	167.18 ± 8.84	185.94	0.04
16	CSB 600 - m3	10.07 ± 0.05	0.50 ± 0.01	8.94 ± 0.04	28.03 ± 0.34	6.91 ± 0.11	70.70 ± 0.34	3.32 ± 0.23	16.88 ± 0.38	0.41 ± 0.02	0.49 ± 0.02	151.68 ± 10.58	69.58 ± 3.77	212.53	0.04

The biochars had a wide array of characteristics, which may help to understand the mechanisms of Cd adsorption. Eight biochars were produced by slow pyrolysis, which is characterized by a higher residence time of the biomass inside the reactor (hours or days), while some were produced by fast pyrolysis with a shorter retention time (within minutes), and some for non-pyrolysis methods (i.e., gasification or thermal carbonization by flame). We did not observe a clear relationship between the production method (and residence time inside of the reactor) and biochar properties. Thus, feedstock seems to have played a more important role than production method, as showed by the literature. Gao et al. (2019) showed that rice straw biochars retain more Cd than sewage sludge biochars. Bair et al. (2016) found greater Cd retention with walnut shell biochar at 900 °C, than softwood biochar at 510 °C. Poultry litter biochar (400 °C) retained more Cd than eucalyptus biochar (600 °C) (Lu et al., 2014, 2015), and *Sida hermaphrodita* biochar adsorbed 13% more Cd than the wheat straw biochar due to greater surface functional groups content (Bogusz et al., 2015).

The biochars were mostly alkaline, except SWB 650 and SSB 450, which had pH of 6.88 and 6.47, respectively (Table 2). The production temperature alone did not seem to influence biochar pH, as we found that samples ASB 500-1 and SSB 450 had respective pH of 10.52 and 6.47. However, within the same feedstock, the increased production temperature led to an increase in pH from 6.47 to 12.53 for SSB 450 and SSB 700, respectively. When using elevated temperatures there is usually an increase in pH because of the increase in basic cations in the ash, associated with alkaline species, such as carbonates (Houben et al., 2013) and a reduction in acidic functional groups (Yuan et al., 2011).

In terms of EC, SSB 700 and WSB 900 were moderately saline with EC of 6.86 and 6.02 mS cm^−1^ respectively, while SWB 800 – i and SWB 500, ASB 500–1 and 2, SSB 450 and WSB 700–60 are slightly saline (EC ranging from 2-4 mS/cm) and the rest are non-saline with EC < 2 mS cm^−1^ in analogous comparison to soil salinity and crop growth tolerance classification (Tanji and Kielen, 2002). When comparing SSB 450 with SSB 700, the increase in the temperature produced a relatively large increase in EC value from 2.84 to 6.86.

Even though each biochar was air-dried until the weight stabilized, moisture content varied widely. CSB 600 - m1 had the highest moisture content, probably due to its particle size distribution (Table 1 -SI), with a large portion of the particles <0.5 mm (96.9%). Ash and Volatile Matter (VM) are important parameters for predicting biochar behavior in soil (Deenik et al., 2011; Wan et al., 2014). Previous research has reported a correlation between high ash content (usually rich in potassium, calcium, and carbonate), high EC, and alkaline pH (Domingues et al., 2017; Tang et al., 2013). Our results suggest that the feedstock played a more important role than ash content because SSB 450 had the second highest ash content of 59.69%, a moderate EC and the lowest pH of 6.47. The typically high ash content of sewage sludge biochar may be explained by its high metal content (Paz-Ferreiro et al., 2018) and to soil contamination (usually containing 1–2% dry solids) during production, which increases its mineral content (Hong et al., 2009). The volatile matter (VM) content is related to the level of thermal carbonization of biochars, while the percentage of VM corresponds to the non-carbonized (labile) domain and percent remaining in the carbonized fraction or fixed carbon (Antal and Grønli 2003). These studies have shown a correlation between VM and lower production temperature. Our highest VM contents were from biochar produced at temperatures between 500 and 600 °C, with CSB 600 and its first two modified versions, and SWB 500 and ASB 500–1 having VM contents >28%.

Most biochars had a C content above 60%, but SSB 450, SSB 700 and CSB 600–m2 had C contents of 23.3, 26 and 51.2, respectively. As expected, sewage sludge biochars had a low C content, ranging from 15 to 40% of total C depending on the pyrolysis temperature, which is consistent with previous work (Agrafioti et al., 2013; Zhao et al., 2014; Pituello et al., 2015; Waqas et al., 2014). CSB 600 – m2 had its C content reduced from its original biochar during the proprietary modification, but had higher VM content. For plant-derived biochars, which are enriched in aromatic C (Wiedemeier et al., 2015), Cd binds better to low charge-highly carbonized biochars by electrostatic attraction with biochar π electrons. Additionally, Cd diffusion into pores may be facilitated by an increase in surface area and pore volume (Chen et al., 2012). In these cases, biochar's CEC decreases probably due to the loss of O-containing functional groups (Melo et al., 2013).

Further, sewage sludge biochars had the highest concentration of N and S, and increased production temperature increased the S content from 3.72 to 6.45% and decreased N content from 3.25 to 1.54% in SSB 450 and SSB 700, respectively. Biochars with S contents higher than the detection limit, but lower than 1%, included CSB 600 and its modified versions and WSB 900. All other biochars had less than 1% N. Sulfur, as a heavier atom, tends to concentrate with increasing production temperatures (Melo et al., 2013). Corroborating this idea, Chen et al. (2019) chemically modified rice husk biochars with abundant amino and disulfide functional groups and noticed a 10-fold increase in Cd retention compared to the unmodified biochar. Different results were presented by Cantrell et al. (2012), Gaskin et al. (2008) and Knudsen et al. (2004), who showed thermal decomposition of inorganic S and volatilization of S-containing volatile organic compounds, which decreased the S content as a result of increasing temperature.

Biochars with low H/C and O/C were WSB 900 and WSB 700-60 and WSB 700-90. Sewage sludge biochars had the highest O/C, N/C and S/C content, but not a higher CEC. Lower H/C values tend to correlate to a higher aromaticity and lower (N + O + S)/C ratio to a higher polarity (Bourke et al., 2007). In fact, biochars with higher CEC content were WSB 900, SWB 800 – i, CSB 600 m2, WSB 700–60 and WSB 700–90 corresponding to 203.57, 176.65, 167.18, 164.08 and 145.62 cmolc kg^−1^, respectively. In general, there is a slight correlation between high production temperature and higher CEC (Singh et al., 2010), as with SSB 450 and SSB 700, the CEC decreased from 53.92 and 44.86 cmol_c_ kg^−1^ as production temperature increased. These sewage sludge biochars had some of the lowest CEC values from the dataset. In contrast, Singh et al. (2010) found that biochars made from manure, paper sludge, and poutry litter had a higher CEC than biochars made from wood and leaves.

As expected, DOC values generally were correlated with VM content. For example, the highest DOC values came from biochars with highest VM. Exceptions were SSB 450, with a high content of inorganic volatile compounds, and CSB 600 – m1, with a high VM content and a relatively low DOC content.

No general trend was observed among biochars and total pore area or volume. Biochars with the highest total pore area were SWB 800 – i, WSB 900, WSB 700–60, and WSB 700–90, showing again that feedstock may play a more important role than production temperature in determining porosity. Total pore volume directly correlated with total area, and among the four biochars cited, SWB 800 – i and WSB 700–60 had the highest volume of 0.08 cm^3^ g^−1^, followed by the other two, with 0.07 cm^3^ g^−1^.

**Figure 1:**
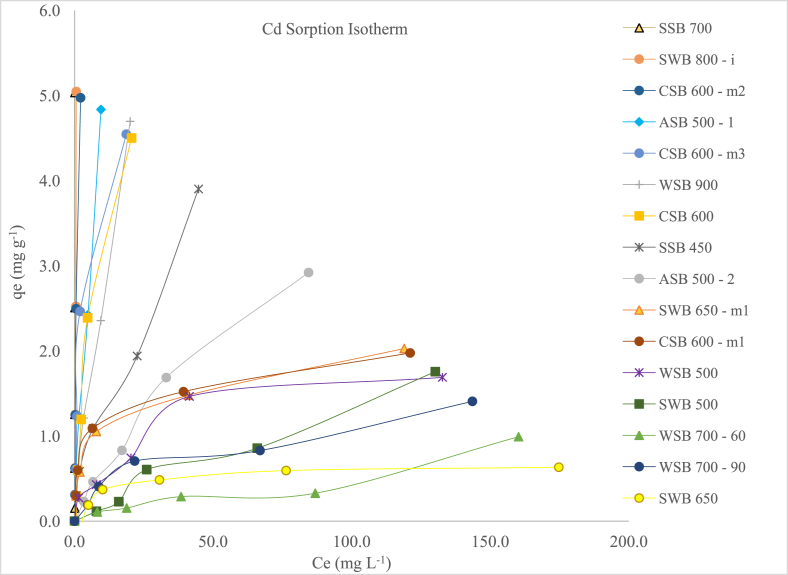
Biochar-Cd adsorption isotherms for un-aged biochars.

The FTIR spectra for all biochars are presented in Figure 1 -SI. Some biochars, such as SSB 700, WSB 700–90, WSB 700–60, WSB 900, ASB 500–1 and SWB 800 – i, had a high aromatic content, with very few surface functional groups. Biochars with numerous surface functional groups showed an absorbance peak around 3540 cm^−1^ attributed to O–H, and the bands 3110-3000 cm^−1^ (aromatic C–H), 3000-2800 cm^−1^ (aliphatic C–H), 1765-1700 cm^−1^ (aromatic carbonyl/carbonyl C=O), 1650-1600 cm^−1^ (aromatic C=C and/or carboxylate C–O and or conjugated ketone C=O), 1470-1370 cm^−1^ (aliphatic C–H), 1160-100 (ester, phenol C–O–C, C–OH), 975-700 cm^−1^ (aromatic C–H). SSB 450 showed a peak in the region of 1190-1127 cm^−1^ (alcohol, ether, phenol C–O–C, poly O–H) and around 670 attributed to O–H as proposed by Parikh et al. (2014) and Silverstein et al. (2014).

### 3.2 Cd adsorption isotherms for un-aged biochars

The results for the sixteen unaged biochar-Cd adsorption isotherms are presented in Figure 1. The y-axis represents the adsorption capacity at equilibrium in mg kg^−1^ and the x-axis the Cd concentration in solution after reacting for 24 h. Two types of isotherms can be differentiated according Giles et al. (1960). CSB 600, CSB 600 – m2 and ASB 500–2 corresponding to type “L”, which indicates that there is a high affinity for the biochar surface at low concentrations of the adsorbate, but this affinity decreases as coverage increases. All the others biochars presented an isotherm with an “H” shape, which is an extreme version of the L-type and is characterized by a high slope at low concentrations and a plateau for high concentrations (Essington, 2004). To further understand the mechanisms involved in the Cd adsorption by biochars, each isotherm was adjusted with the most common adsorption models: Langmuir and Freundlich models.

Additionally, three groups of retention can be identified: 1) high retention, with a steep slope and no plateau: SSB 700, SWB 800 – i, CSB 600 – m2, ASB 500–1, CSB 600 – m3, WSB 900 and CSB 600; 2) medium retention, with a lesser slope and no plateau: SSB 450, ASB 500 -2; and 3) lower retention and reaching a plateau: CSB 600 – m1, WSB 500, SWB 500, WSB 700–60, WSB 600–90 and SWB 650. Group 1 was selected for further examination by aging (leaching biochars with H_2_O_2_) with the intention of further maximizing polar functional groups and thus maximizing Cd retention.

### 3.3 Adsorption models

Langmuir and Freundlich model parameters are presented in Table 3, as well as the percentage of Cd retention for each biochar. The Langmuir model allowed us to estimate the values for the adsorption maxima (*Q*_*max*_) and measure of the adsorption intensity (*K*_*L*_). The Freundlich model allowed us to estimate *K*_*F*_ (kg L^−1^) and *1/n* (unitless) related to adsorption capacity and adsorption intensity, respectively. The Freundlich model stipulates that the adsorption capacity decreases logarithmically as the surface becomes covered by the adsorbate (Echeverría et al., 1998).

**Table 3:** Biochar-Cd adsorption models result.

#	Sample	Langmuir			Freundlich			Retention
		Q_max_ (mg g^−1^)	K_L_ (L mg^−1^)	R^2^	1/*n*	Log k_*F*_	R^2^	(%)
1	SWB 800 – i	31.71	65.95	0.66	1.83	5.73	0.98	99.69
2	SWB 650	0.67	0.04	1.00	0.31	0.75	0.85	12.62
3	SWB 650 – m1	2.10	0.58	0.99	0.34	1.28	0.97	40.51
4	SWB 500	8.58	0.14	0.19	0.94	1.16	0.96	34.91
5	ASB 500 - 1	8.55	8.15	0.79	0.76	2.42	0.99	95.23
6	ASB 500 - 2	5.84	0.40	0.91	0.79	1.54	0.99	57.78
7	WSB 500	1.96	0.18	0.76	0.39	1.15	0.96	33.62
8	WSB 900	10.44	4.07	0.80	0.82	2.24	0.98	89.96
9	WSB 700 - 60	1.63	0.01	0.30	0.69	0.71	0.91	19.88
10	WSB 700 - 90	1.65	0.07	0.92	0.52	1.05	0.92	28.21
11	SSB 450	4.62	1.31	0.78	0.55	1.69	0.99	77.62
12	SSB 700	415.67	113.30	0.60	15.80	46.56	0.98	99.92
13	CSB 600	5.49	8.07	0.98	0.80	2.30	0.94	89.71
14	CSB 600 - m1	2.02	0.78	0.99	0.31	1.28	0.94	39.44
15	CSB 600 - m2	15.36	55.97	0.23	0.93	3.35	0.89	98.91
16	CSB 600 - m3	7.84	4.26	0.89	0.62	2.22	0.87	90.67

Due to the heterogeneous set of biochars we studied, the Q_max_ results varied from 0.67 to 415.67 mg g^−1^, but, in general, all biochars sorbed Cd; see Table 3. Both models were able to represent the adsorption phenomenon. In contrast to some literature (e.g., Li et al., 2020), the Freundlich model provided a better fit for the adsorption isotherms than the Langmuir model (higher R^2^ values). This is probably due to the heterogeneity of the biochar surface because Cd-biochar interaction probably does not occur in a monolayer, or because some of the isotherms did not reach a plateau. We speculate that a retention plateau might have been reached for the high-retention biochars if we had used less biochar (we suggest 0.12 g, rather than 0.48 g).

When analyzing the results of Q_max_ from the Langmuir model, the biochar adsorption capacity order did not exactly follow the three groups of retention observed from the isotherms: 1) high retention *Q*_*max*_ > 10 mg g^−1^: SSB 700, SWB 800 – i, CSB 600 – m2, and WSB 900; 2) medium retention *Q*_*max*_ 10-5 mg g^−1^: SWB 500, ASB 500 - 1, and CSB 600 – m3, ASB 500 -2, and CSB 600; 3) low retention *Q*_*max*_ 5-0 mg/g: SSB 450, CSB 600 – m1, WSB 500, WSB 700–60, WSB 600–90 and SWB 650. For example, SSB 450 clearly had a steeper isotherm than ASB 500–2, but the former had a Q_*max*_ of 4.62 and ASB 500 -2 of 5.84. Or, CSB 600 -m1 would be expected to have a higher Q_*max*_ than SWB 500, but the former and latter had Q_*max*_ of 2.02 and 8.58, respectively.

Additionally, Worch (2012) stated that if 1/n > 1, then the isotherm is considered favorable, that is, the adsorbate has higher affinity for the adsorbent than for the solution. If 1/n = 1 the isotherm is linear and considered type C (Essington, 2004). If 1/n < 1, then the isotherm is considered unfavorable. In this case, we have two groups: 1) favorable isotherms that include SSB 700 and SWB 800 – i; and 2) unfavorable isotherms that include SWB 500, CSB 600 – m2, WSB 900, CSB 600, ASB 500–2, ASB 500–1, WSB 700–60, CSB 600 – m3, SWB 450, WSB 700–90, WSB 500, SWB 650 – m1, SWB 650 and CSB 600 – m1. This means that SSB 700 and SWB 800 – i have a greater affinity for adsorbing Cd, but there is no practical implication of this affinity in regards to someone choosing a biochar to help remediate Cd may be cited, because we have data from desorption experiments (not shown) and biochars with unfavorable adsorption isotherms had similar desorption isotherms as SWB 800-i. When determining retention %, the distribution of biochar's groups coincides with the isotherm groups and it seems to be better explanation of the results. Because of this, we decided to use retention data in the correlations.

### 3.4 Correlation between Cd retention and biochar properties

**Figure 2:**
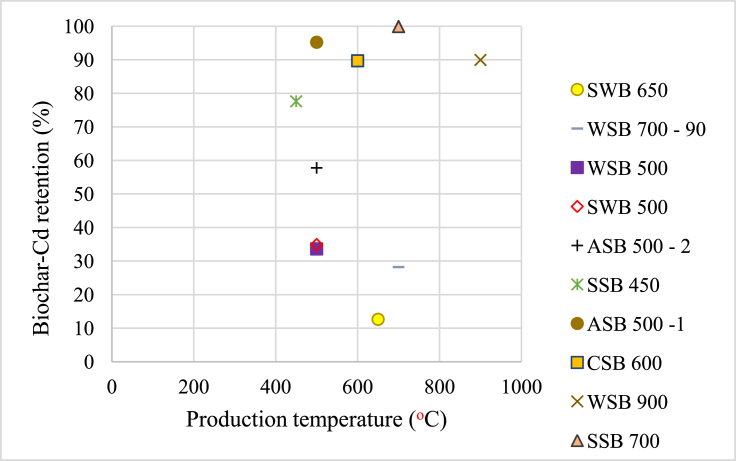
Impact of production temperature of non-aged and non-modified biochars on Cd retention.

For the analysis of the correlation between biochar properties and Cd retention, we excluded pH, moisture content, and total pore volume from the analysis. The variable pH in water was removed because all biochars-Cd isotherms had solution pH adjusted to 7. Moisture content and total pore volume were excluded because they are independent variables. Moisture content was measured by heating samples at 105 °C and it had a statistical influence on every other property because each property was measured on air-dried biochar. Total pore volume is a variable dependent of total area of pores (TAP). Principal components analysis (PCA) yielded Table 2 -SI and Figure 2 – SI.

Following PCA, we fit a linear regression model using the four main components from the PCA matrix predicting Cd retention (%). Component 1 is statistically significant (P = 0.014), and the model explains 46.4% of the variance with 11 degrees of freedom (R adjusted, n < 30). Components 2, 3 and 4 showed no statistical significance (P > 0.01).

The analysis revealed that the properties that played more important roles in Cd retention on component 1 (the component that better explained the model) were directly attributed to ash (0.910), S% (0.904), and N% (0.824), and inversely to C% (−0.911). Similar results were found by Deng et al. (2020), where mineral co-precipitation was foremost for high temperature biochars. Confirming our hypothesis, O% did not play such a significant role as the literature suggests (Zhu et al., 2020). As discussed by Yuan et al. (2020), surface area and pore volume also did not play a significant role as originally expected and reported elsewhere (e.g., Sizmur et al., 2017). These results show that Cd retention is the result of a combination of factors and may be much more complex than what the literature has been suggesting. Wan et al. (2020) suggest measuring electrochemical properties by cutting-edge technologies, such as X-ray diffraction (XRD, crystalline and interlayer spacing), Fourier transform infrared spectrometry (FTIR, functionalities), X-ray photoelectron spectroscopy (XPS), Raman spectroscopy, elemental analyzer (EA, ultimate elemental analysis), scanning electron microscopy (SEM, surface morphological images) coupled with X-ray disperse spectroscopy (EDS, proximate elemental analysis) and transmission electron microscopy (TEM, high-resolution morphology, pore diameter determination, and crystalline spacing) etc., to understand biochar behavior.

Additionally, the model separated the samples into two groups related to Cd retention. We tried to identify a categorizing property or a combination of properties (e.g., fixed carbon, H/C, N/C, O/C) that would explain this separation, increase R^2^ and, thus, increase the prediction of retention. However, no clear trend was found with the properties we measured. Other properties might include anion exchange capacity, alkalinity, redox potential, aromaticity, degree of aromatic condensation.

Finally, Cd retention could be significantly enhanced after N or S surface functionalization. For instance, many authors employed a facile ball-milling method to successfully dope N basic sites on the inert and negatively charged surface of biochar using kinetic energy of moving balls (Kumar et al., 2020c). Another approach could be making biochar with ammonia (NH_3_) ambiance. NH_3_ may react with oxygen-containing species of biomass to form N-containing heterocyclic compounds (i.e., kenones, aldehydes, esters, furans) (Wang et al., 2020b).

### 3.5 Correlation between pyrolysis temperature and Cd retention

Biochar production temperature has been shown to affect Cd retention. In general, an increase in temperature increases Cd retention for biochars made with the same feedstock. For example, Harvey et al. (2011), Kim et al. (2013), Melo et al. (2013), Cui et al., 2016a, Cui et al., 2016b, Zhou et al. (2018), Liu et al. (2019), and Wang et al. (2019), studied a thermosequence of plant-derived biochars. These papers reported an increase in Cd retention with an increase in production temperature. Increased Cd retention was attributed to (i) an increase in C and decrease in hydrogen (H) and oxygen (O) content, leading to an increase in biochar aromaticity and, consequentially, a decrease in polar functional groups; and (ii) an increase in surface area and pore volume.

Biochars made from sewage sludge at 500, 600, 700, 800 and 900 °C also resulted in increased Cd retention with increasing temperature, but it was primarily due to an increased surface area and CEC (Chen et al., 2014). One reason for why CEC played a more important role for these biochars is that sewage sludge biochars can be relatively rich in N (up to 2.8%) and S (up to 2.3%) (Paz-Ferreiro et al., 2018; Waqas et al., 2015). Additionally, Shen et al. (2019) demonstrated that increasing temperature decreased exchangeable Cd fraction immobilized on biochar, which may be representative in sewage sludge biochar.

Figure 2 shows the impact of production temperature on un-aged biochars. We present data only for non-modified biochars and excluded CSB 600-m1, -m2 and -m3 and SWB 800 - i. We also found this correlation among biochars made from the same feedstock and production method, e.g., SSB 450 and SSB 700.

However, among different feedstocks and different production methods, we found no clear correlation between pyrolysis temperature and Cd retention. This could be explained by a production method issue, wherein the temperature reported by each company may vary from actual production temperatures. The company may report the reactor target temperature or the reactor atmosphere temperature, but not the exact temperature inside the feedstock. Also, the retention time is very important for the feedstock to reach the target temperature. Depending on how the company measured the temperature, the real temperature of the feedstock may be different.

For example, biochars ASB 500 - 1 and ASB 500 - 2 had the same feedstock and were made at the same pyrolysis temperature, but by different companies, and they had very different retention rates. In this case, the companies may have reported a pyrolysis temperature taken from different places of the reactor, or the production method plays a more important role than expected. On the other hand, ASB 500–1 and WSB 500 were produced by the same company under the same temperature, and ASB 500–1 had a much higher retention than WSB 500, which would suggest that for the same temperature, almond shell biochars would retain more Cd than walnut shell biochars. The same case can be observed for CSB 600 and SWB 650, two biochars produced by the same company that had a very different retention rate and almost the same production temperature, in this case we conclude that coconut shell biochars retain more Cd than softwood biochars. However, studies with a larger dataset are needed to confirm this conclusion.

### 3.6 Aging effects

**Figure 3:**
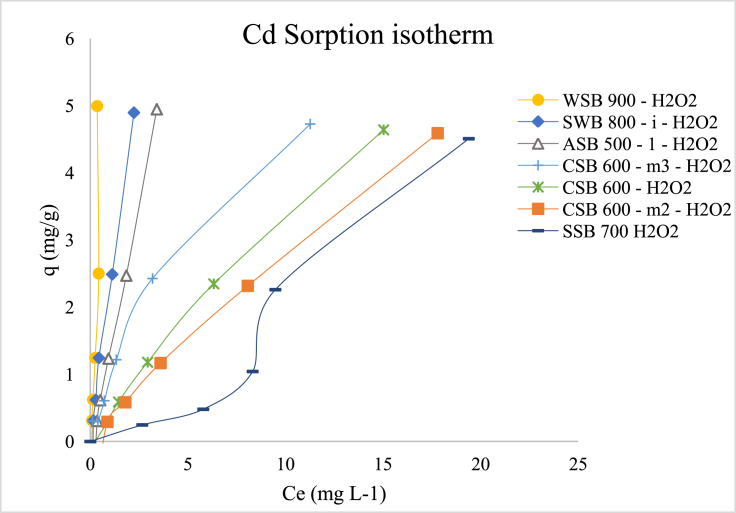
Biochar-Cd adsorption isotherm for aged biochars.

The Cd-biochar isotherms for the biochars oxidized with H_2_O_2_ are shown in Figure 3. Our results indicate that the isotherms were quite similar in terms of shape to their non-aged version, being classified as H-type, except for SSB 700 H_2_O_2_, which presented an S-type. The order of retention was: WSB 900 H_2_O_2_ > SWB 800 – I H_2_O_2_ > ASB 500–1 H_2_O_2_ > CSB 600 H_2_O_2_ > CSB 600 – m2 H_2_O_2_ > SSB 700 H_2_O_2_.

The oxygenation of the biochar surface was done to maximize Cd retention, following literature recommendations. It was expected that H_2_O_2_ would increase CEC due to the addition of acidic oxygen functional groups on the surface of the biochar (Huff and Lee 2016) and thus, increase cation sorption efficiency (Frišták et al., 2015; Zuo et al., 2016). The results however showed that some of the biochars increased their retention, while others did not (Table 4).

**Table 4:** Correlation between non-aged and aged biochars.

#	Freundlich model	Non-aged			Aged/Oxidized			Δ log K_f_	Δ Retention
	Sample	Log k_*f*_	R^2^	Retention	Log k_*f*_	R^2^	Retention	(%)	(%)
1	SWB 800 – i	5.73	0.98	99.69	3.74	0.95	98.88	-34.73	-0.81
2	ASB 500 - 1	2.42	0.99	95.23	3.42	0.99	98.30	+41.32	+3.22
3	WSB 900	2.24	0.98	89.96	5.69	0.93	99.82	+154.02	+10.96
4	SSB 700	46.56	0.98	99.92	2.79	0.93	90.31	-94.00	-9.62
5	CSB 600	2.3	0.94	89.71	2.45	0.99	92.49	+6.52	+3.10
6	CSB 600 - m2	3.35	0.89	98.91	2.22	0.99	91.10	-33.73	-7.89
7	CSB 600 - m3	2.22	0.87	90.67	2.5	0.98	94.37	+12.61	+4.08

WSB 900 (one of the most aromatic biochars, see Figure 3 - SI), increased its retention by 10.96%, showing that increasing oxygenated functional groups helps with Cd retention. On the other hand, SSB 700, which is also highly aromatic had its retention reduced by 9.62%. This opposite trend may be the result of a more significant reduction in SSB 700 H_2_O_2_ ash content than in WSB 900 (Table 3 - SI), even though O content increased in both.

It was expected that H_2_O_2_ would dissolve part or all of the biochar labile C domain and remove mineral ash from pores, hence, increasing surface area and abundance of oxygenated functional groups (Sizmur et al., 2017). We observed that, independent of the increase in oxygen content in all biochars (Table 3 - SI), the increase in retention was not significant for the majority of the biochars and even reduced the retention in some. This result suggests that Cd use in soils will be a continuous process, as biochar will need to be reapplied to with time even though it is a highly recalcitrant material.

Chemical modifications such leaching with acidic and alkaline solutions mimic this increase in oxygenating surface functional groups (Sizmur et al., 2017). Because modification of biochars with strong acids can be expensive at a large scale and pose environmental concerns because of the disposal of the effluent (Sun et al., 2019), especially among alkali treatments, H_2_O_2_ has been proposed as an popular alternative, for being less expensive and a cleaner product and process (Huff and Lee 2016). However, after the process, a washing procedure is necessary to eliminate the agents applied in impregnation, which limits its application to some level (Kumar et al., 2020b). We recommend the use of H_2_O_2_ only for conceptual studies, such this one.

Finally, in a pot experiment conducted by the same authors of this paper (Alves et al., 2021) where spinach was planted in a Cd-rich soil, the best biochars when considering maximum yield and minimum Cd uptake were WSB 900 and SSB 700 H_2_O_2_ both at 5% v/v application rate. Although this result seems promising, similar yields and uptake was obtained by poultry litter compost at 5% v/v application rate. Considering that compost is usually much cheaper than biochar, biochar application in farmland may still be not feasible. In a cost-benefit analysis, published in 2015, where the use of biochar to improve cereals agriculture was investigated, the prospect of using it was not promising, being a poor investment option for farmers (Dickinson et al., 2015). On the other hand, a more recent research showed that the net benefit over a hectare would amount to US$8581, or US$105 per metric ton of biochar applied (Joseph et al., 2020). Our understanding is that the benefit of using biochar is very biochar specific. Additionally, new production methods are being developed to make biochar more economically feasible (A. Kumar et al., 2020a).

### 3.7 Comparison of adsorption rates with the literature

Lastly, we compared the adsorption results from the current study with the literature (see Table 5). As it can be noticed, the majority of the biochars used were vegetable derived, much fewer were sludge or manure derived. Additionally, when comparing biochars that were modified with their original ones, it is possible to see that not all modifications are successful in maximizing Cd adsorption, that is the case of CSB 600 (Q_max_ = 5.5 m g^−1^) and CSB 600 - m1 (Q_max_ = 2.0 m g^−1^) in this study and BC (Q_max_ = 15.1 m g^−1^) and PBC (Q_max_ = 7.0 m g^−1^) in Zhu et al. (2020). On the other hand, most of the modifications increased Cd adsorption, that is the case of SWB 600 (Q_max_ = 0.7 m g^−1^) and SWB 600 – m1 (Q_max_ = 2.1 m g^−1^) and CSB 600 (Q_max_ = 5.5 m g^−1^) and CSB 600 – m2 (Q_max_ = 15.4 m g^−1^) in this study; CK (Q_max_ = 8.4 m g^−1^) and C_0,75_ (Q_max_ = 81.0 m g^−1^) in Chen et al. (2019) and others. The biggest increase happened in Zhang et al. (2018) where the unmodified biochar BC0c had a Q_max_ = 25.0 m g^−1^ and its Na_2_HPO_4_ modified version had a Q_max_ = 202.6 m g^−1^. Biggest Q_max_ were found on PBC (Q_max_ = 423.0 m g^−1^) which was a biochar made from algae at 500 °C modified by H_3_PO_4_ (Li et al., 2020), on SSB 700 (Q_max_ = 415.7 m g^−1^) (this study) and M85 (Q_max_ = 406.5 m g^−1^) which was a biochar made from rice husks at 800 °C modified by FeSO_4_ and NaOH (Yuan et al., 2020) showing that modification with addition of P and S functional groups to biochar's surface may be promising for maximizing Cd removal. Although, the use of unmodified sewage sludge has led to similar adsorption rates and will be more cost-effective and environmentally friendly. Another thing to point out, is that the three best biochars (PBC, SSB 700 and M85) had temperatures of 500, 700 and 800 °C, while biochars with the lowest Q_max_ also had temperatures ranging from 450 to 700 °C, showing high temperatures do not guarantee Cd retention. Also, 90% of the samples had a Q_max_ ranging from 0.34 to 93.41 m g^−1^, showing great variation among biochars.

**Table 5:** Comparison of different Biochars and adsorbents materials of the Cd adsorption results, using Langmuir and Freundlich.

Biochar	Feedstock	Temperature (°C)	Langmuir			Freundlich			Reference
			Q_max_ (mg g^−1^)	K_L_ (L mg^−1^)	R^2^	Log k_*F*_	1/*n*	R^2^	
SWB 800 – i	Softwood inoculated	800	31.71	65.95	0.66	5.730	1.83	0.98	This study
SWB 650	Softwood	650	0.67	0.04	1	0.750	0.31	0.85	
SWB 650 – m1	Softwood modified by proprietary methods	650	2.1	0.58	0.99	1.280	0.34	0.97	
SWB 500	Softwood	500	8.58	0.14	0.19	1.160	0.94	0.96	
ASB 500 - 1	Almond Shell	500	8.55	8.15	0.79	2.420	0.76	0.99	
ASB 500 - 2	Almond Shell	500	5.84	0.4	0.91	1.540	0.79	0.99	
WSB 500	Walnut Shell	500	1.96	0.18	0.76	1.150	0.39	0.96	
WSB 900	Walnut Shell	900	10.44	4.07	0.8	2.240	0.82	0.98	
WSB 700 - 60	Walnut Shell	700	1.63	0.01	0.3	0.710	0.69	0.91	
WSB 700 - 90	Walnut Shell	700	1.65	0.07	0.92	1.050	0.52	0.92	
SSB 450	Sewage Sludge	450	4.62	1.31	0.78	1.690	0.55	0.99	
SSB 700	Sewage Sludge	700	415.67	113.3	0.6	46.560	15.8	0.98	
CSB 600	Coco nutshell	600	5.49	8.07	0.98	2.300	0.8	0.94	
CSB 600 - m1	Coco nutshell modified by proprietary methods	600	2.02	0.78	0.99	1.280	0.31	0.94	
CSB 600 - m2	Coco nutshell modified by proprietary methods	600	15.36	55.97	0.23	3.350	0.93	0.89	
CSB 600 - m3	Coco nutshell modified by proprietary methods	600	7.84	4.26	0.89	2.220	0.62	0.87	
BCS	Crop residue	650–700	32.57	0.043	0.9896	-1.194	0.84	0.84	Bogusz et al., (2015)
BCSH	Crop residue	700	35.71	0.2	0.9986	-0.921	1.88	0.77	
BCU400	residue of biogas production	400	68.63	0.04	0.9418	-1.201	1.83	0.84	Bogusz et al., (2017)
BCU600	residue of biogas production	600	76.34	0.105	0.9334	-0.680	4.05	0.27	
BCS600	residue of biogas production	600	32.57	0.043	0.9896	-1.194	0.84	0.84	
CK	Rice husk	-	8.35	0.072	0.98	0.337	0.27	0.89	Chen et al., (2019)
C 0.75	Rice husk modified with cystamine dihydrochloride	-	81.02	0.016	0.987	0.564	0.58	0.96	
CIB300	Crop residue	300	63.32	0.32	0.997	1.296	3.85	0.89	Cui. Fang et al., (2016)
CIB400	Crop residue	400	105.78	0.18	0.968	1.465	3.33	0.95	
CIB500	Crop residue	500	188.79	0.53	0.887	1.770	2.94	0.74	
CIB600	Crop residue	600	140.01	1.03	0.876	1.718	3.85	0.74	
TDB	Crop residue	500	58.29	0.211	0.935	1.133	2.73	0.84	Cui. Hao et al., (2016)
PAB	Crop residue	500	30.98	0.042	0.949	0.479	0.49	0.99	
VZB	Crop residue	500	37.27	0.014	0.977	0.041	0.67	0.97	
ZCB	Crop residue	500	36.7	0.259	0.971	0.972	0.34	0.96	
RB400	rice straw. washed with HCl	400	16.14	-	-	-	-	-	Deng et al., (2020)
RB700	rice straw. washed with HCl	700	48.65	-	-	-	-	-	
HRB400	rice straw. aged after incubations and washed with HCl	400	14.97	-	-	-	-	-	
HRB700	rice straw. aged after incubations and washed with HCl	700	32.23	-	-	-	-	-	
BCA	beech wood chips	500	1.99	0.07	-	-0.699	0.59	-	Frišták et al., (2014)
BCB	garden green waste residues	500	7.8	0.08	-	-0.222	0.87	-	
AC	activated carbon	-	2.62	0.63	-	-0.921	0.71	-	
BC350	Crop residue	350	55.5	0.003	0.84	36.560	3.41	0.98	Goswami et al., (2016)
BC400	Crop residue	400	71.43	0.003	0.95	45.190	4.03	0.97	
BC500	Crop residue	500	62.5	0.001	0.95	55.100	4.15	1.00	
BC550	Crop residue	550	41.67	0.001	0.9	119.390	4.19	0.96	
ABC	activated biochar	-	72.43	0.001	0.941	163.300	4.57	0.95	
OWB	Oak wood char	400–450	0.37	377	0.5748	-0.638	8.33	0.58	Mohan et al., (2007)
PBB	Pine bark char	400–450	0.34	2	0.7427	-0.398	2.86	0.78	
OBB	Oak bark char	400–450	5.4	55	0.7723	0.528	1.02	0.85	
Carbon F-400	activated carbon	-	8	4859	0.9141	0.822	14.3	0.93	
BC300	Crop residue	300	11.4	0.383	0.97	0.663	0.24	0.96	Kim et al., (2013)
BC400	Crop residue	400	11.99	0.506	0.95	0.714	0.24	0.98	
BC500	Crop residue	500	13.24	2.329	0.98	0.903	0.17	0.97	
BC600	Crop residue	600	12.96	10.51	0.9	0.955	0.15	0.99	
PBC	Algae modified with H3PO4	500	423	3	0.99	0.350	1.27	0.97	Li et al., (2020)
KBC	Algae modified with KMnO4	600	142	4	0.71	0.528	1.85	0.81	
ZBC	Algae modified with ZnCl2	500	–	5.24 × e−4	0.84	-2.999	0.46	0.99	
TMBC	Sycamore tree sawdust aminothiourea chitosan modified magnetic biochar composite	550	93.72	6.596	0.9957	0.955	0.51	0.83	Li et al., (2018)
BC	Rape straw	600	32.737	0.142	0.989	0.768	2.45	0.97	Li et al., 2017a, Li et al., 2017b)
BC-FeOx	Rape straw modified with FeOx	600	67.363	0.085	0.985	0.939	2.12	0.96	
BC-MnOx	Rape straw modified with MnOx	600	81.096	0.09	0.994	0.952	1.82	0.99	
BC-NaOH	Rape straw modified with NaOH	600	72.369	0.102	0.993	0.988	1.87	0.98	
PS	Pine sawdust biomass	-	3.47	0.079	0.996	-0.138	0.39	0.93	Liu et al., (2019)
PSB-500	Pine sawdust	500	4.78	0.145	0.999	0.225	0.38	0.92	
PSB-700	Pine sawdust	700	6.09	0.342	0.999	-0.851	0.3	0.87	
PBC700	pig manure	700	92.68	-	-	-	-	-	Wang et al., (2018)
BBC700	bamboo	700	77.08	-	-	-	-	-	
CBC700	corn straw	700	76.18	-	-	-	-	-	
BC250	aquatic plant (water hyacinth)	250	49.5	0.131	0.81	-	-	-	Zhang et al., (2015)
BC350	aquatic plant (water hyacinth)	350	69	0.137	0.96	-	-	-	
BC450	aquatic plant (water hyacinth)	450	70.313	0.11	0.949	-	-	-	
BC550	aquatic plant (water hyacinth)	550	34	1.135	0.756	-	-	-	
-	mango peel waste	-	67.08	0.085	0.998	0.799	0.45	0.95	Iqbal et al. (2009)
PHs	peanut husk	-	26.88	0.01	0.901	1.407	0.32	0.92	Cheng et al., 2016
PHB	peanut husk biochar	500	28.99	0.7961	0.9722	1.796	0.22	0.98	
-	Active carbon	-	11.27	0.02	0.96	-0.215	0.53	0.94	Ulmanu et al., (2003)
-	Kaolin	-	3.04	0.07	0.85	-0.108	0.28	0.94	
-	Bentonite	-	9.27	22.7	0.97	0.270	0.47	0.98	
-	Diatomite	-	3.24	0.36	0.72	0.250	0.14	0.89	
-	Compost	-	9.34	0.68	0.98	0.579	0.33	0.96	
-	Anaerobic sludge	-	–	–	–	-0.602	1.59	0.96	
-	Cellulose pulp waste	-	5.82	0.05	0.99	-0.046	0.37	0.98	
MSB300	maize straw	300	30.3	0.03	0.93	0.481	0.41	0.97	Wang et al., (2019)
MSB400	maize straw	400	30.12	0.09	0.97	0.723	0.35	0.90	
MSB500	maize straw	500	35.46	0.08	0.94	0.793	0.35	0.90	
MSB600	maize straw	600	17.21	0.04	0.98	0.504	0.29	0.98	
PLB300	Platanus leaves	300	21.83	0.03	0.97	0.519	0.32	0.97	
PLB400	Platanus leaves	400	14.16	0.45	0.99	0.876	0.12	0.82	
PLB500	Platanus leaves	500	25.45	0.12	0.97	1.009	0.16	0.97	
PLB600	Platanus leaves	600	19.49	0.13	0.99	0.948	0.13	0.99	
M85	Rice husks modified by FeSO4 and NaOH	800	406.46	-	-	-	-	-	Yuan et al., (2020)
pm-BC3c	bamboo modified by Na2HPO4	750	202.55	0.09	0.972	1.313	0.24	0.89	Zhang et al., (2018)
BC0c	bamboo	750	24.95	0.04	0.983	0.656	0.28	0.96	
BC	corn stalk	600	15.14	0.72	0.92	0.843	0.23	0.99	Zhu et al., (2020)
PBC	corn stalk modified by K2CO3	600	7.02	0.04	0.98	-0.237	0.55	0.95	
APBC	corn stalk modified by HNO3and NH3	600	23.54	0.04	0.995	0.305	0.53	0.99	
OPBC	corn stalk modified by HNO3	600	19.04	0.99	0.965	0.974	0.21	0.99	

Finally, comparing biochar with the use of non-pyrolyzed feedstocks, biochar in general had a higher adsorption capacity than active carbon, kaolin, bentonite, diatomite, compost, anaerobic sludge and cellulose pulp waste. Although mango peel waste seems to be a promising adsorption material.

## 4 Conclusions

This research evaluated the ability of different kinds of biochars to adsorb Cd, and the mechanisms involved, by understanding the role of specific biochar properties in Cd retention. It is known that these properties vary depending on the feedstock and the pyrolysis conditions, but how they vary is not yet clear. Hence, the study of a wide variety of biochars helped us to identify major trends in biochar Cd retention relative to biochar properties. We did not observe a clear relationship between the production method (and retention time) and biochar properties. We observed a slight positive relationship between production temperature and Cd retention, but in general, feedstock type seems to play a much more important role in determining Cd retention.

Two types of isotherms were observed for biochar-Cd retention (L and H-type curves), while the retention dynamics occurred at three levels, and with or without plateaus. Both Langmuir and Freundlich models were able to represent the adsorption phenomenon, but Freundlich fit the curves better, probably due to the heterogeneity of the biochar surface, and because some of the isotherms did not reach a plateau. We recommend repetition of these experiments for high retention biochars with a smaller quantity of biochar (0.12 g instead of 0.48 g, for a higher solution to soil ratio).

The biochar properties that played the most important roles in Cd retention were ash, S%, N% and C%. Our hypotheses: (i) that biochars with greater soft base functional group contents (S + N) will bind Cd more effectively than others biochars that have higher O-containing functional groups, and (ii) for biochars poor in S and N functional groups, C content will play a bigger role in Cd retention than surface area and CEC; were confirmed. Our results show that Cd retention occurs by a combination of factors. However, we recommend future studies with an increase of the number of samples and properties analyzed. We also recommend a development of an algorithm to try to understand the combination of these mechanism to be able to predict biochar's behavior. For that, we recommend the use of cutting-edge technology to elucidate Cd-sorption mechanisms.

The aging experiments showed that even though the O content increased in all biochars, the increase in retention was not significant for the majority of the biochars and even reduced the retention in some. This result suggests that Cd use in soils will be a continuous process, as biochar will need to be reapplied to with time even though it is a highly recalcitrant material. Data from this study can be used to design biochars with maximized sites for binding with soft acids such as Cd, by providing guidance for manufacturers (e.g., to produce an N- and S- enriched biochar) or to identify a post-production modification that can be used to enhance these functional groups (e.g., chemical treatment). Additionally, considering that sludge solids generated in the European Union, China and the USA are at 10 Mt, 39 Mt and 7.2 Mt, respectively (Kumar et al., 2021), we also hope to stimulate the usage of sewage sludge biochar as a waste management solution.

## Declarations

### Author contribution statement

Bárbara Samartini Queiroz Alves: Conceived and designed the experiments; Performed the experiments; Analyzed and interpreted the data; Contributed reagents, materials, analysis tools or data; Wrote the paper.

Luiz Arnaldo Fernandes: Conceived and designed the experiments; Performed the experiments; Analyzed and interpreted the data.

Randy Southard: Contributed reagents, materials, analysis tools or data; Wrote the paper.

### Funding statement

This work was supported by Coordenação de Aperfeiçoamento de Pessoal de Nível Superior - CAPES agency and the Brazilian federal government with the Science Without Borders Scholarship. Additional funds were provided by a Henry A. Jastro Research Award, an SBG Student Fellowship award, and the biochar company, Cool Planet.

### Data availability statement

Data included in article/supplementary material/referenced in article.

### Declaration of interests statement

The authors declare no conflict of interest.

### Additional information

No additional information is available for this paper.

## Appendix A Supplementary data

PAPER 1_SI PAPER 1_SI
