# Supplementary material for: Biochar-cadmium retention and its effects after aging with Hydrogen Peroxide (H2O2)
Source: Heliyon. 2021 Nov 26;7(12):e08476. doi: 10.1016/j.heliyon.2021.e08476 (PMC8649738; doi:10.1016/j.heliyon.2021.e08476)
Supplement: PAPER 1_SI [file mmc1.docx]

**Supporting Information (SI) Of “Biochar-Cadmium Retention and its Effects After Aging with Hydrogen Peroxide H_2_O_2_”**

Table S1 - Particle size distribution of unaged biochars.

| # | Sample | **Particle size distribution (%)** | | |
| --- | --- | --- | --- | --- |
|  |  | **<0.5mm** | **0.5-1 mm** | **1-2mm** |
| 1 | SWB 800 – i | 48.68 | 15.02 | 36.30 |
| 2 | SWB 650 | 9.34 | 22.45 | 68.21 |
| 3 | SWB 650 – m1 | 39.83 | 20.51 | 39.66 |
| 4 | SWB 500 | 35.56 | 27.26 | 37.18 |
| 5 | ASB 500 - 1 | 62.55 | 17.98 | 19.47 |
| 6 | ASB 500 - 2 | 50.07 | 22.98 | 26.95 |
| 7 | WSB 500 | 29.50 | 21.72 | 48.78 |
| 8 | WSB 900 | 35.65 | 17.05 | 47.30 |
| 9 | WSB 700 - 60 | 43.68 | 19.40 | 36.92 |
| 10 | WSB 700 - 90 | 46.02 | 18.23 | 35.75 |
| 11 | SSB 450 | 46.88 | 24.23 | 28.89 |
| 12 | SSB 700 | 32.14 | 16.90 | 50.96 |
| 13 | CSB 600 | 23.80 | 15.15 | 61.05 |
| 14 | CSB 600 - m1 | 16.27 | 29.10 | 54.63 |
| 15 | CSB 600 - m2 | 96.90 | 1.10 | 2.00 |
| 16 | CSB 600 - m3 | 16.25 | 26.10 | 57.65 |

Figure S1 - DRIFT spectra of un-aged biochars.

| **Table S2 – Principal component matrix.**   \|  \| Component \| \| \| \| \| --- \| --- \| --- \| --- \| --- \| \| 1 \| 2 \| 3 \| 4 \| \| EC \| .745 \| .161 \| -.465 \| .005 \| \| VM \| .106 \| .064 \| .132 \| .978 \| \| Ash \| .910 \| -.313 \| -.140 \| .128 \| \| N \| .824 \| -.184 \| -.004 \| .008 \| \| C \| -.911 \| .177 \| -.250 \| -.084 \| \| H \| -.481 \| .139 \| .826 \| .072 \| \| O \| .084 \| .203 \| .859 \| .068 \| \| S \| .904 \| -.290 \| -.125 \| -.037 \| \| DOC \| -.219 \| 0.958 \| .122 \| .035 \| \| CEC \| -.225 \| .957 \| .124 \| .041 \| \| TAP \| -.613 \| 0.059 \| -.612 \| -.304 \| |   **Figure S2 - Principal Components graph**. |
| --- | --- | --- | --- | --- | --- | --- | --- | --- | --- | --- | --- | --- | --- | --- | --- | --- | --- | --- | --- | --- | --- | --- | --- | --- | --- | --- | --- | --- | --- | --- | --- | --- | --- | --- | --- | --- | --- | --- | --- | --- | --- | --- | --- | --- | --- | --- | --- | --- | --- | --- | --- | --- | --- | --- | --- | --- | --- | --- | --- | --- | --- | --- | --- | --- | --- |

Figure S3 - DRIFT spectra of aged biochars.

Table S3 - Characterization for aged biochars.

| Sample | pH | EC | Moist. | VM | Ash | C | H | O | N | S |
| --- | --- | --- | --- | --- | --- | --- | --- | --- | --- | --- |
|  |  | (mS/cm) | % | | | | | | | |
| SWB 800 - i H2O2 | 9.78 | 0.61 | 16.89 | 17.59 | 13.39 | 71.07 | 2.33 | 18.60 | 0.55 | 0.05 |
| ASB 500 H2O2 | 6.72 | 0.35 | 42.38 | 42.53 | 3.75 | 72.91 | 3.36 | 23.30 | 0.22 | 0.02 |
| WSB 900 H2O2 | 10.31 | 0.93 | 28.63 | 18.43 | 10.93 | 81.10 | 2.49 | 18.80 | 0.47 | 0.02 |
| SSB 700 H2O2 | 6.83 | 2.32 | 7.28 | 21.37 | 67.71 | 21.70 | 1.24 | 18.90 | 1.35 | 4.20 |
| CSB 600 H2O2 | 4.67 | 0.23 | 8.27 | 32.75 | 1.57 | 67.04 | 3.31 | 21.40 | 0.44 | 0.02 |
| CSB 600 - m2 H2O2 | 4.02 | 0.17 | 5.97 | 33.22 | 11.23 | 59.05 | 3.12 | 21.00 | 0.94 | 0.04 |
| CSB 600 - m3 H2O2 | 6.42 | 0.14 | 7.08 | 29.74 | 5.53 | 67.90 | 3.12 | 18.70 | 0.86 | 0.07 |
